# Pupil Tracking Without Goggles: A Solar-Assisted TinyML Brim-Based Pupil and Head Motion Tracking System with Digital Mimic

**DOI:** 10.3390/mi17101183

**Published:** 2026-10-13

**Authors:** Robert Graziano, Linda Valitutti, Juliette Bassett, Anwar Elhadad

**Affiliations:** Thomas J. Watson College of Engineering and Applied Science, Binghamton University, State University of New York, Binghamton, NY 13902, USA

**Keywords:** wearable sensors, sensor applications, sensor data processing, sensor interface electronics, sensor system integration, sensor system networks

## Abstract

This work presents a solar-assisted wearable pupil-tracking microsystem that integrates on-device inference, head pose sensing, energy harvesting, and mobile visualization within a single hat-based platform. The device is fabricated using 3D-printed thermoplastic polyurethane (TPU), providing a flexible and durable structure that supports embedded electronics while maintaining user comfort. The brim houses two ESP32-S3 microcontrollers, two grayscale eye-facing cameras, an inertial measurement unit (IMU), a lithium-polymer battery, a custom-shaped flexible amorphous silicon photovoltaic module, and a charging circuit. Each microcontroller runs an INT8-quantized convolutional neural network (CNN) that performs real-time 2D pupil coordinate regression and eye closure classification from 64 × 64 grayscale images. Pupil coordinates, eye state information, and head pose measurements are transmitted via Bluetooth Low Energy (BLE) to a custom iOS application, where a Unity-based 3D avatar mirrors the wearer’s relative eye movement and head motion while enabling lightweight data logging for offline analysis. By relocating sensing, computation, and power electronics from eyewear to the hat brim, the proposed system enables unobtrusive, autonomous pupil and eye state monitoring for naturalistic cognitive and behavioral research without requiring personal point-of-gaze calibration.

## 1. Introduction

Eye tracking has become an increasingly important sensing modality for understanding human attention, cognition, and behavior [1,2]. Measurements such as fixation duration, saccadic motion, blink frequency, and gaze transitions have been shown to correlate with cognitive workload, visual attention, neurological disorders, and mild cognitive impairment (MCI) [3]. As a result, eye-tracking systems are widely used in psychology, neuroscience, human–computer interaction (HCI), augmented reality (AR), virtual reality (VR), driver monitoring, and clinical assessment [4,5,6]. Recent studies show that gaze behavior can serve as a non-invasive biomarker for cognitive decline and neurological disease, further motivating the development of wearable eye-tracking technologies that operate in natural environments [7]. Current eye-tracking systems generally use image-based pupil detection, in which one or more cameras capture images of the eye and software algorithms estimate gaze direction from pupil location [8,9]. While highly accurate laboratory systems exist, they typically require fixed camera installations and controlled lighting conditions that restrict user mobility [10]. Wearable eye trackers address these limitations by positioning cameras near the eye, enabling continuous monitoring during everyday activities [11,12]. Commercial systems such as Tobii Pro Glasses and Pupil Labs Core have demonstrated the value of mobile eye tracking in real-world environments; however, they largely rely on eyeglass-based architectures that constrain sensing, computation, communication, and power integration within the limited volume of a glasses frame [13,14].

The physical constraints imposed by eyewear present several challenges. First, the limited space available within eyeglass frames restricts battery capacity, embedded processing resources, and sensor placement. Consequently, many wearable eye trackers rely on tethered operation, external processing devices, or bulky hardware modules positioned near the face [15]. Second, cameras and supporting electronics mounted directly on the user’s face may introduce discomfort and alter natural behavior, particularly for individuals who do not routinely wear glasses [16]. Finally, the proximity of the sensors to the eye creates challenges related to occlusion, mechanical alignment, and long-term wearability, limiting deployment in unconstrained environments [17].

Recent advances in embedded machine learning, low-power microcontrollers, wireless communication, and flexible energy-harvesting technologies create opportunities for alternative wearable architectures that move beyond traditional eyewear-based designs [18,19]. Rather than further miniaturizing glasses-mounted systems, a larger wearable substrate can be utilized to distribute sensing, computation, communication, and power-management functions while maintaining user comfort. Such an approach enables local edge processing, reduces communication bandwidth requirements, and provides additional volume for energy storage and harvesting components. To address these limitations, this work presents a solar-assisted wearable eye-tracking microsystem integrated into a 3D-printed thermoplastic polyurethane (TPU) hat. The proposed platform utilizes the brim of the hat as a structural substrate for integrating sensing, computation, wireless communication, and energy harvesting. Two brim-mounted grayscale cameras capture images of the user’s eyes from an unobtrusive upward viewing angle, while dedicated ESP32-S3 microcontrollers execute INT8-quantized convolutional neural networks that estimate normalized pupil coordinates and eye closure state directly on-device. The system further incorporates an inertial measurement unit (IMU) for head pose estimation, Bluetooth Low Energy (BLE) communication for real-time data transmission, and a custom-shaped flexible amorphous silicon photovoltaic module integrated into the hat structure for energy harvesting and autonomous operation. Unlike conventional wearable eye trackers that stream raw video for external processing, the proposed system performs inference directly on the embedded hardware and transmits only low-dimensional gaze and head pose information. The resulting data are visualized through a mobile application that drives a real-time digital avatar while simultaneously enabling lightweight data logging for offline analysis. By combining distributed sensing, embedded artificial intelligence, wireless communication, and solar-assisted power management within a single wearable platform, the proposed architecture provides an unobtrusive and autonomous alternative to traditional eyewear-based eye-tracking systems.

### 1.1. System Architecture

**Figure 1:**
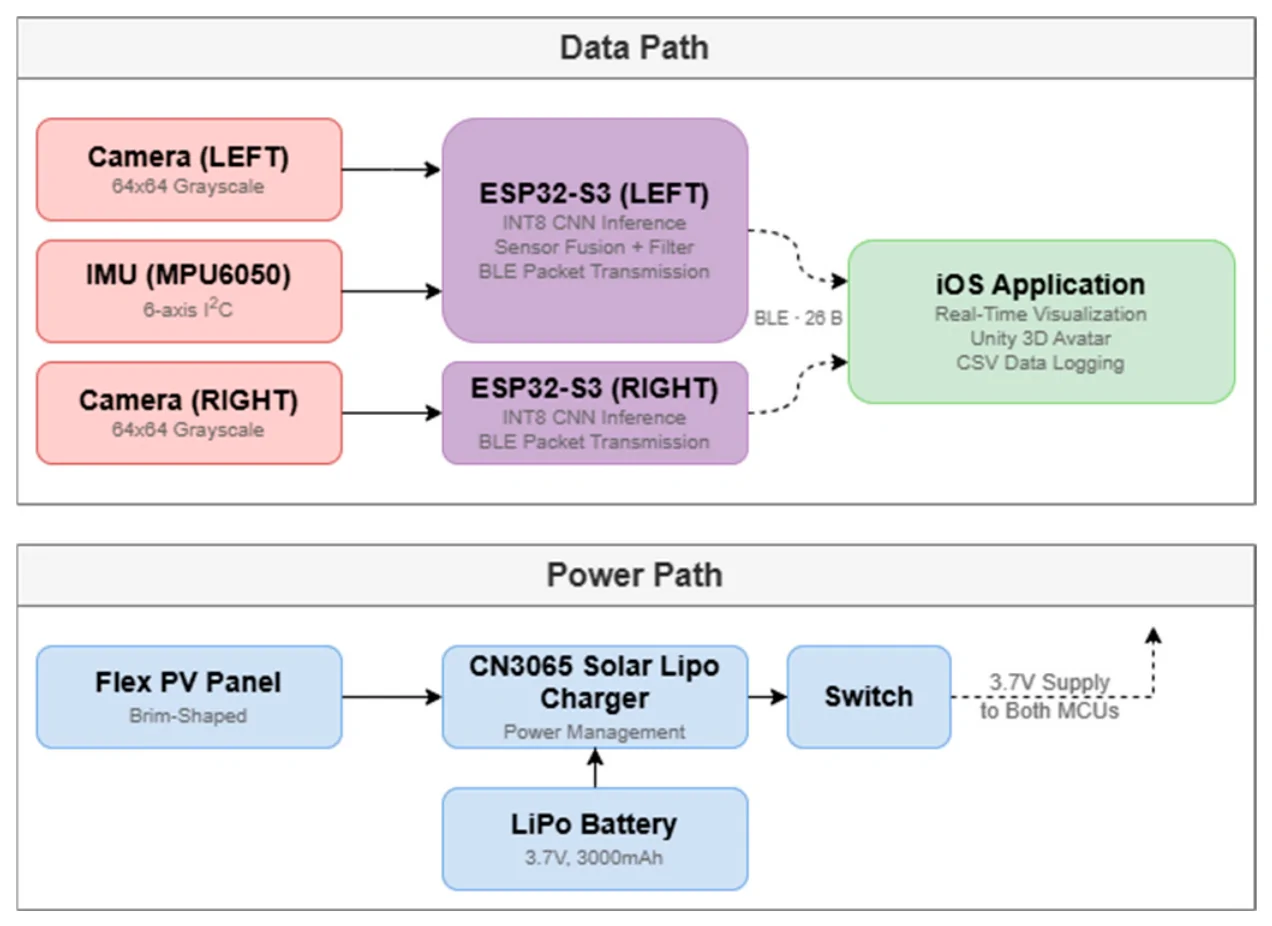
Flowchart of the system architecture, containing the data path and the power path.

The proposed eye-tracking microsystem integrates sensing, embedded intelligence, wireless communication, and energy harvesting within the structural volume of a 3D-printed TPU hat. As illustrated in Figure 1, the system is organized into two tightly coupled subsystems: a data path responsible for gaze sensing, embedded inference, and wireless communication, and a power path responsible for energy harvesting, storage, and regulation. Together, these subsystems enable fully autonomous eye tracking without external processing hardware or tethered power sources.

#### 1.1.1. Data Path

The data path consists of two primary sensors, the microcontrollers, the wireless communication, and the IOS application. Two primary sensors are distributed across the brim, each serving a distinct role in gaze sensing. The primary sensors are two grayscale, three-megapixel CMOS image sensors, namely, OV3660, integrated with the ESP32 modules. These sensors have a field of view (FOV) of 68°, which is narrow enough to include just the eye in the frame of the image. This provides an advantage when measuring gaze, as the pupil will take up more of the frame, allowing the algorithm to detect smaller differences. The cameras are operated with automatic exposure control to preserve robustness against varying ambient illumination, and each frame is histogram-equalized in firmware before being downsampled from 96 × 96 to 64 × 64. The secondary sensor is a six degrees of freedom (DOF) inertial measurement unit (IMU) that features a three-axis gyroscope and three-axis accelerometer. This is connected to the left microcontroller via the I^2^C bus. The IMU data is sampled at the same rate as the image pipeline to synchronize the pupil and head movement. Since both cameras are mounted to the same brim, only one of the microcontrollers needs to be processing the IMU data. The estimated yaw and accelerometer-derived absolute roll and pitch are sent through the BLE data packet along with the eye-tracking data.

**Figure 2:**
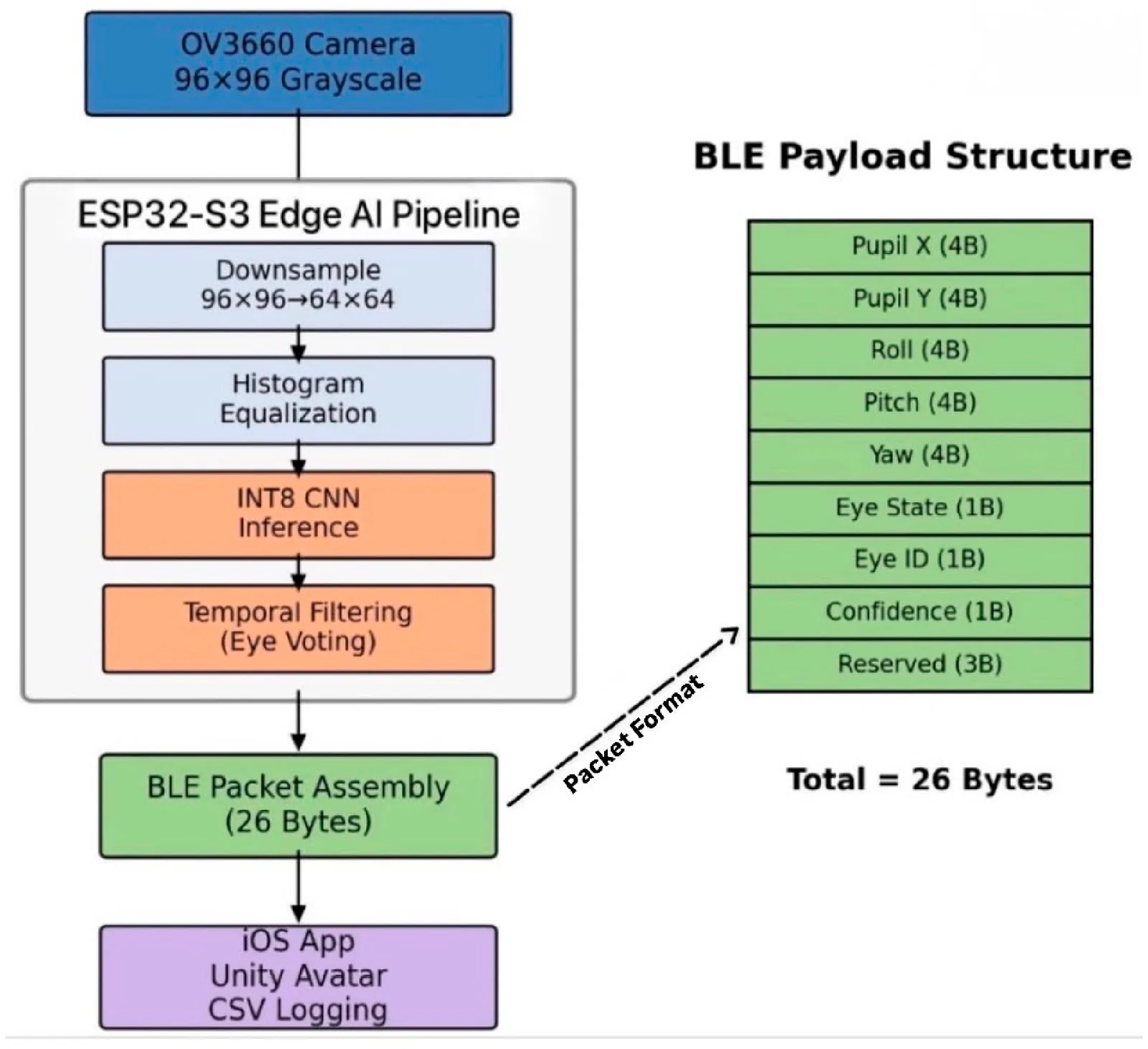
Embedded edge AI processing and communication pipeline. Estimated gaze coordinates and eye state information are packaged into a 26-byte BLE payload and transmitted to the iOS application for real-time visualization and logging.

The data processing is built around two ESP32-S3 microcontrollers, each dedicated to a single eye. Although it would be possible to use one processor for both image streams, there are substantial advantages to separating them. Firstly, the system employs two ESP32-S3 microcontrollers rather than a centralized processing architecture. This distributed configuration increases image-processing throughput, enables independent optimization of the left- and right-eye models, and reduces computational contention between simultaneous image streams. In order to capture as many frames as possible, we use the double frame buffer possible via the ESP32-S3’s PSRAM. This allows for a frame to constantly be ready to be processed without needing to wait for an image to be captured. Secondly, since both eyes are not perfectly symmetrical, we can deploy separate models on each of the microcontrollers tailored to the specific eye it is capturing. To minimize on-device storage use, the model is quantized to INT8 precision to reduce memory by four times relative to the float32 baseline. The resulting model is then converted to a C header file via xxd -i and compiled directly in firmware, occupying 167 KB. Additionally, the model’s tensor arena is allocated in external PSRAM to preserve the constrained internal DRAM for the wireless stack and operating system. The embedded processing and communication pipeline is illustrated in Figure 2. Each 96 × 96 grayscale image acquired by the OV3660 camera is downsampled to 64 × 64 pixels and histogram-equalized prior to INT8 CNN inference. The resulting pupil coordinates are temporally filtered using pupil smoothing and eye presence voting before being assembled into a compact 26-byte BLE payload. The packet contains gaze coordinates, IMU-derived head pose estimates, eye state information, and confidence metrics for transmission to the mobile application.

The IOS application subscribes to ESP32-S3 microcontrollers through custom BLE GATT services using notify characteristics. This architecture allows the left and right sensing nodes to connect and disconnect independently while maintaining synchronized gaze acquisition. Received packets are parsed and forwarded to a Unity-based avatar, where eye movements and head rotations are rendered in real time. The application additionally supports CSV logging for lightweight storage and subsequent replay of gaze behavior without requiring video recording.

#### 1.1.2. Power Path

**Figure 3:**
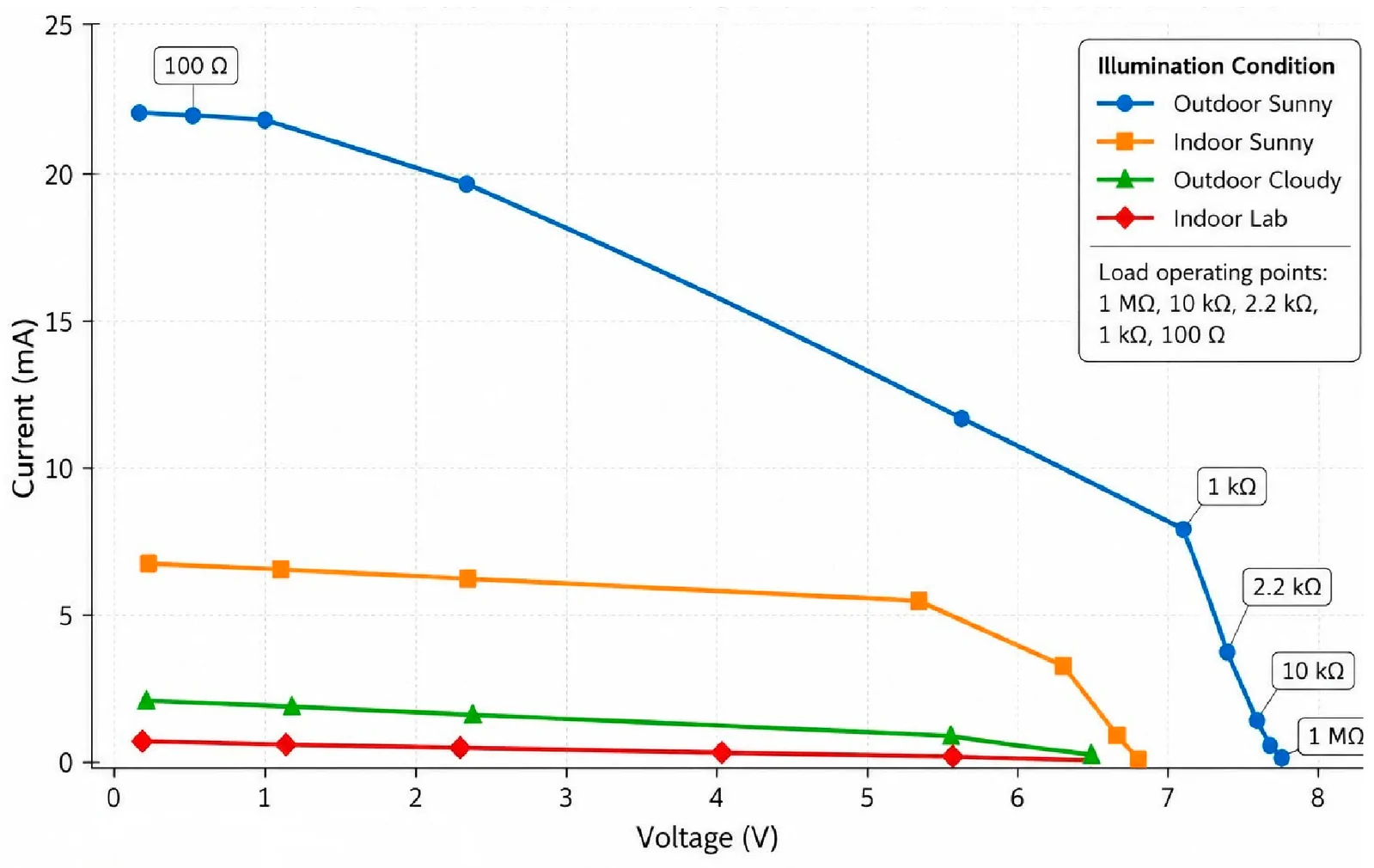
Electrical characterization of the custom-shaped flexible amorphous silicon photovoltaic module integrated into the hat.

The power subsystem enables energy-autonomous operation through the integration of a custom-shaped flexible amorphous silicon photovoltaic (PV) module, a 3000 mAh Li-ion battery, and an embedded charging circuit. Unlike conventional wearable eye trackers that rely exclusively on battery power, the proposed architecture harvests ambient light energy to extend operating lifetime and reduce charging frequency. The PV module is fabricated to conform to the curvature of the hat brim and interfaces directly with a Li-ion charging IC that manages energy harvesting and battery charging. Electrical characterization of the PV module under outdoor sunny, indoor sunny, outdoor cloudy, and laboratory lighting conditions is shown in Figure 2. The complete sensing, embedded inference, and wireless communication pipeline consumes approximately 0.144 A during operation. Powered by the 3000 mAh battery, the system can operate for over 20 h without recharging, while harvested solar energy further extends operational lifetime during outdoor use. A mechanical power switch isolates the electronics during inactive periods while allowing the battery to continue charging. By combining distributed sensing, embedded edge AI inference, wireless communication, and photovoltaic energy harvesting within a single wearable platform, the proposed system forms a compact solar-assisted microsystem capable of long-duration gaze monitoring in natural environments. Figure 3 presents the measured current–voltage characteristics of the custom-shaped flexible amorphous silicon photovoltaic module under multiple illumination conditions. As expected, the highest output voltage and current were observed under direct outdoor sunlight, while reduced power generation was measured under cloudy and indoor lighting conditions. Despite the lower illumination levels, the module continued to generate usable electrical power, demonstrating its ability to supplement battery operation across a range of realistic environments.

## 2. Hardware Design

The hardware is separated into two parts: the mechanical integration and the power electronics.

### 2.1. Mechanical Integration

**Figure 4:**
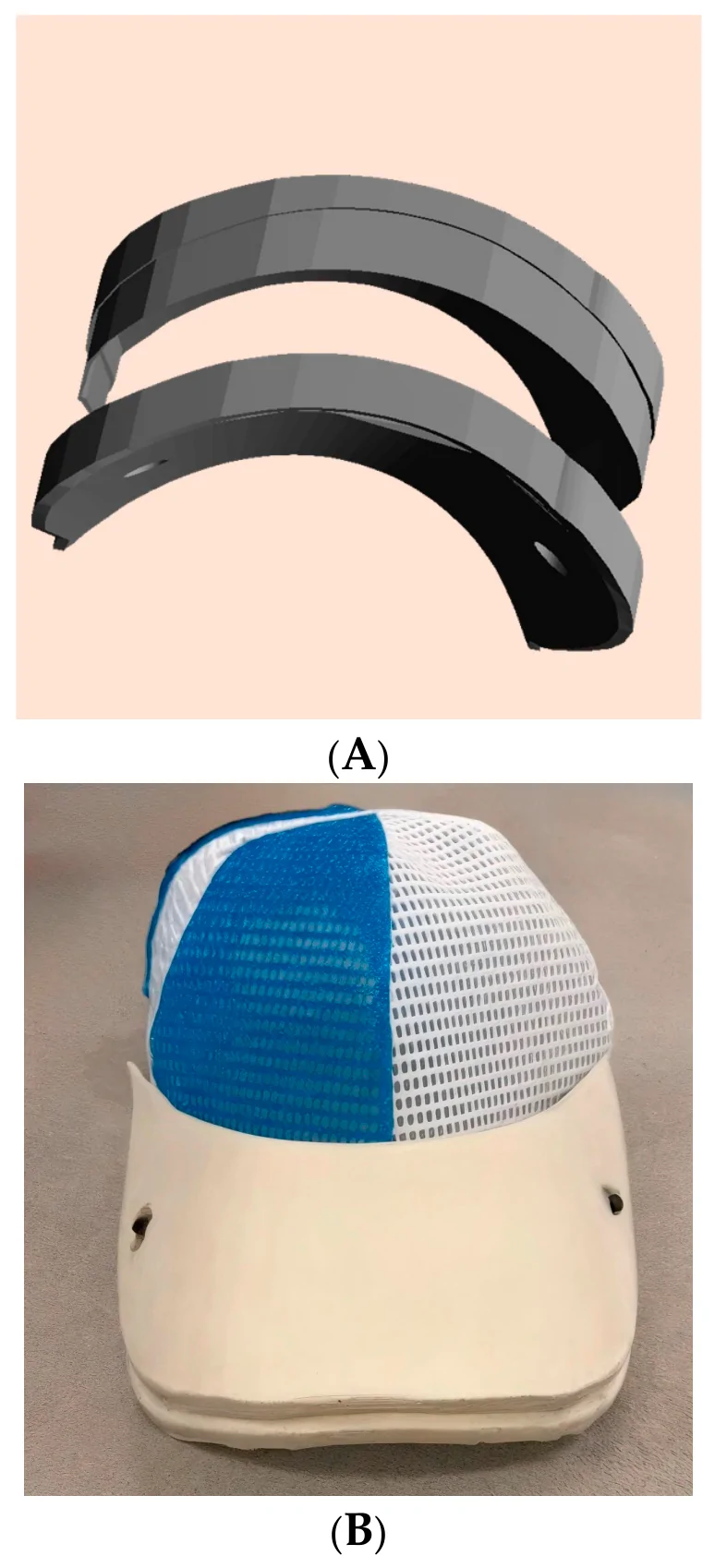
(**A**) 3D exploded view rendering of the top and bottom sections of the brim. (**B**) 3D-printed part that covers the head, attached to the bottom section of the brim.

Figure 4A presents an exploded-view rendering of the brim assembly, illustrating the integration and placement of the embedded electronic components within the wearable structure. The wearable enclosure was fabricated using thermoplastic polyurethane (TPU) to provide a lightweight, flexible, and durable structure suitable for long-term wear, as illustrated in Figure 4B. The brim was designed as a two-piece assembly consisting of upper and lower sections that mechanically interlock, enabling non-destructive access to the embedded electronics for hardware. Supplementary Figure S1 illustrates the physical integration strategy used to embed the sensing, processing, and power subsystems within the wearable platform. The flexible photovoltaic module (Figure S2) occupies the interior dome region to maximize available surface area for energy harvesting while maintaining the external appearance of a conventional hat. The Li-ion battery and power management electronics are housed within the thickened brim structure, enabling distributed packaging of the power subsystem without increasing facial loading. The eye-facing camera is recessed into the underside of the brim, allowing unobstructed observation of the eye while remaining visually concealed. This distributed integration approach leverages the larger volume available in a hat compared with conventional eyewear-based systems, enabling the incorporation of embedded processing, wireless communication, energy storage, and energy harvesting within a single wearable platform. The 92 g electronics payload (dual ESP32-S3 MCUs, dual OV3660 cameras, MPU6050 IMU, solar charger, flexible PV module, and 3000 mAh battery) yields a total hat weight of 192 g. This is comparable to standard headwear (85–115 g) while eliminating the facial contact and nose bridge fatigue typical of smart eyewear. Using a complementary filter, MPU6050 head pose tracking achieved pitch and roll accuracy of 1.1° MAE against an optical reference, with an uncalibrated yaw drift of 0.8°/min. The rigid TPU brim maintains camera alignment during locomotion, restricting displacement jitter to under 0.5 pixels RMS while walking. Under illumination ranging from 500 lux (indoor lab) to 100,000 lux (direct sunlight), preprocessing via histogram equalization maintained stable pupil localization, with error increasing by less than 0.25 pixels across extremes (MAE of 1.42 pixels at 500 lux vs. 1.65 pixels at 100,000 lux). Supplementary Table S4 compares the system against commercial and research eye trackers across cost, calibration burden, weight distribution, and energy harvesting.

### 2.2. Power Electronics

**Figure 5:**
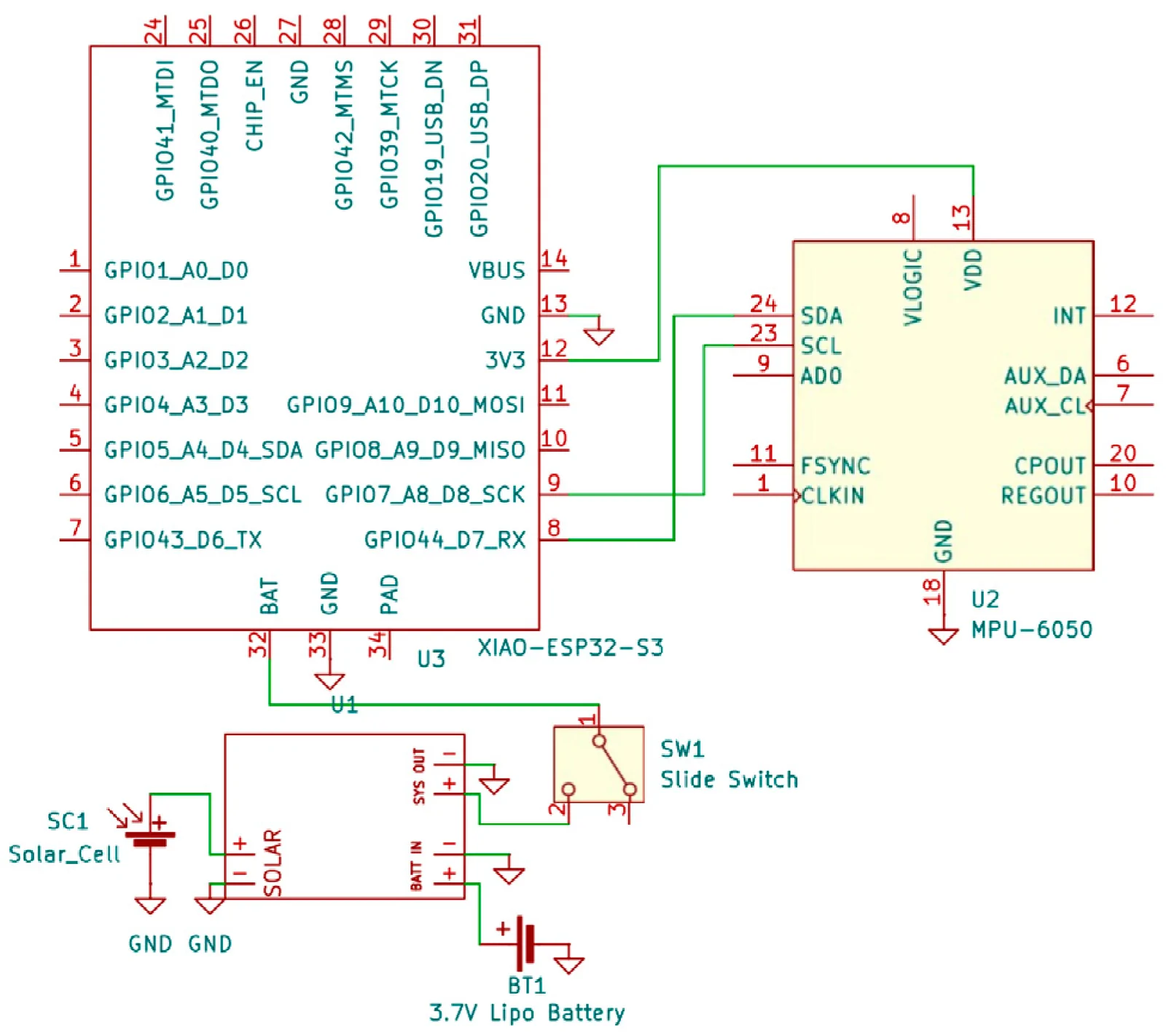
System-level electrical schematic of the proposed wearable eye-tracking platform.

Figure 5 shows the circuit diagram for the left eye’s power electronics embedded inside the brim. The only difference between the right and left eye’s schematics is that the right eye does not have the MPU-6050 accelerometer. As shown, the PV panel and LiPo battery are connected directly to the charger IC. The output of the charger IC is then connected to the slide switch, which connects to the battery pad on the XIAO ESP32-S3. For the left eye, the microcontroller supplies a 3.3V rail to the MPU-6050, and the serial data (SDA) and serial clock (SCL) are connected from the MPU-6050 to the microcontroller’s GPIO 44 and seven pins accordingly.

To evaluate system energy dynamics, a complete power budget was established by benchmarking active hardware power consumption against photovoltaic (PV) generation derived from experimental characterization. The power management system combines a brim-shaped flexible amorphous silicon PV panel, a CN3065 solar LiPo charging manager, and an integrated 3.7 V, 3000 mAh lithium-polymer battery (11.1 Wh). Under continuous active operation (dual-eye INT8 CNN inference, vector extension acceleration, PSRAM buffering, and 26-byte BLE streaming), total active system power consumption is 791.3 mW (Table S3). The two ESP32-S3 microcontrollers constitute the dominant load at 660.0 mW combined, while the dual OV3660 camera sensors and MPU6050 IMU consume 118.8 mW and 12.5 mW, respectively.

## 3. Embedded Machine Learning Pipeline

This section outlines the embedded machine learning pipeline implementation. It is separated into four sections: (A) Dataset Collection, (B) CNN Architecture, (C) Training Procedure, and (D) Quantization and Deployment.

### 3.1. Dataset Collection

The eye-tracking dataset was collected using the brim-mounted custom eye-tracking dataset comprises 3669 grayscale images (96 × 96 pixels downsampled to 64 × 64 for pipeline processing) captured via the brim-mounted OV3660 cameras across four co-author participants during varied ambient illumination and gaze trajectories. The dataset maintains a balanced distribution between eye open (*N* = 2752, 75%) and eye closed (*N* = 917, 25%) states. To prevent temporal data leakage caused by highly correlated neighboring video frames, evaluation was performed using a strict session- and participant-grouped split rather than random frame sampling. The dataset was partitioned at the recording-session level into training (70%, *N* = 2568), validation (15%, *N* = 550), and held-out test (15%, *N* = 551) sets. Ground-truth labels include normalized 2D pupil center coordinates [*x*, *y*] ∈ [0, 1] and binary eye closure state [0, 1]. Public Benchmark Justification: Existing public eye-tracking datasets (e.g., GI4E, LPW) were excluded from formal cross-dataset benchmarking because their images are captured from frontal or eyeglass-mounted perspectives. These perspectives differ substantially in optical geometry, pupil occlusion, and illumination vectors compared to the steep downward view of the brim-mounted cameras used in this platform.

### 3.2. CNN Architecture

**Figure 6:**
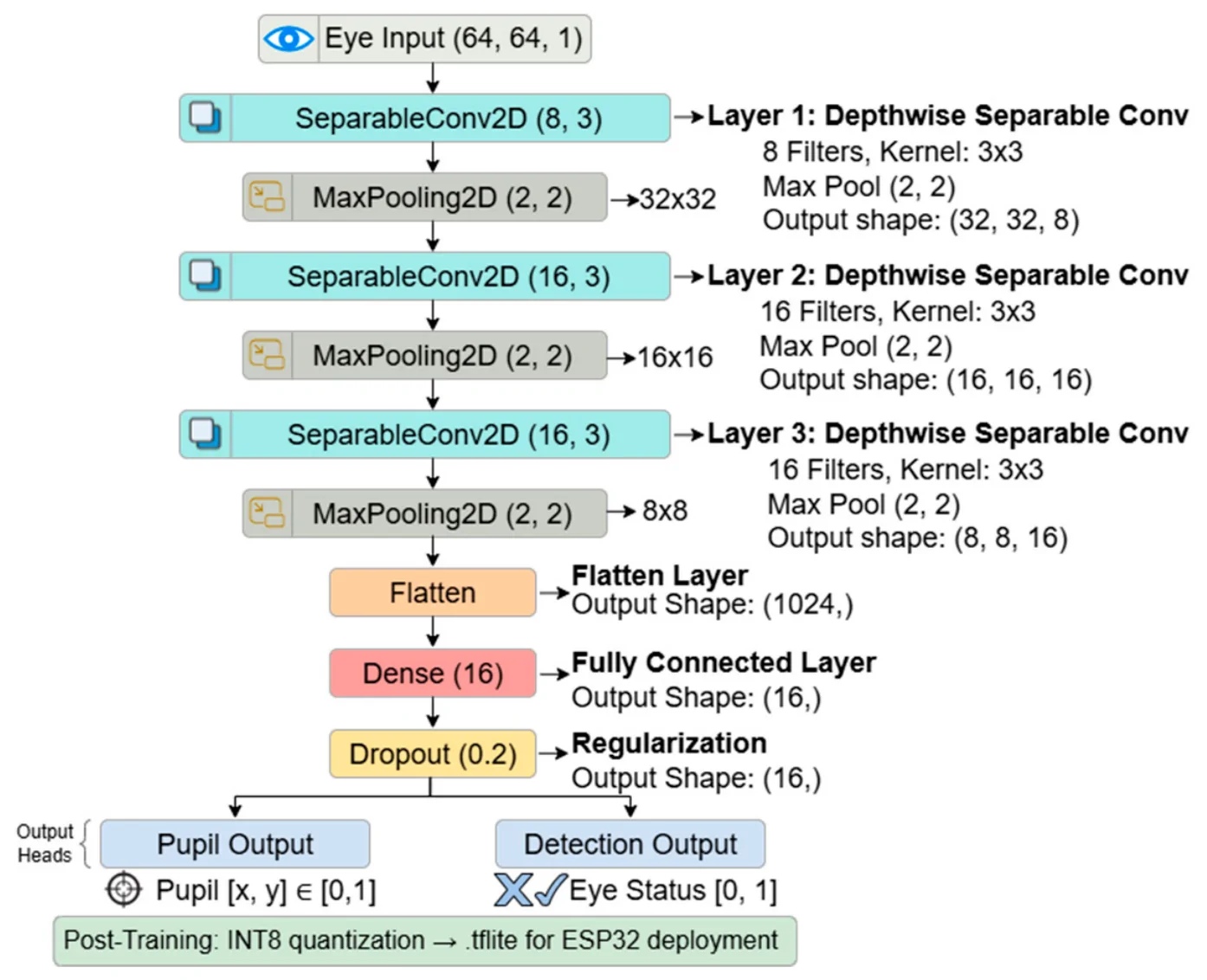
INT8-quantized CNN architecture deployed on the ESP32-S3 for real-time gaze estimation and eye state classification. The network processes 64 × 64 grayscale eye images and outputs normalized pupil coordinates and eye presence predictions.

A lightweight convolutional neural network (CNN) was developed for deployment on the resource-constrained ESP32-S3 microcontroller. The architecture consists of three depthwise-separable convolutional blocks followed by a fully connected feature layer and dual output heads for pupil coordinate regression and eye presence classification. Figure 6 illustrates the network architecture and feature map dimensions throughout the model. Depthwise-separable convolutions were selected to significantly reduce the number of trainable parameters and computational complexity compared to standard convolutions while preserving sufficient feature extraction capability for pupil localization. The use of three convolutional blocks was empirically chosen to balance model accuracy, memory footprint, and inference latency, enabling real-time execution on the embedded platform.

For pupil coordinate regression, Huber loss was employed due to its robustness to annotation noise while maintaining sensitivity to small localization errors. Eye presence classification was trained using binary cross-entropy (BCE) loss, which is well suited for binary classification tasks with sigmoid outputs. The total training objective was formed as a weighted sum of the regression and classification losses, with the eye presence loss scaled by a factor of 0.5 to balance its contribution relative to the coordinate regression task. Following training, the model was quantized to INT8 precision to reduce memory requirements and enable efficient real-time inference on the ESP32-S3.

### 3.3. Training Procedure

The training procedure is as follows. The dataset images are captured in 96 × 96 grayscale. These images are then down-sampled to 64 × 64 and have histogram equalization applied. This histogram equalization is the exact same method used in the ESP32-S3’s inference pipeline. This prevents any lighting invariance between the live and training images, which improves the model’s performance. To prevent closed-eye or invalid coordinate frames from biasing coordinate regression toward the mean label values of the training set, spatial pupil loss is gated by a binary visibility mask m_i_ in {0, 1}. For frames where the eye is open (m_i_ = 1), coordinate loss is calculated normally; for closed-eye frames (m_i_ = 0), coordinate loss is zeroed out, directing gradient updates exclusively through the eye presence classification head. Adaptive learning rate scheduling (ReduceLROnPlateau, halving the learning rate after three epochs of non-improvement) and early stopping (patience of six epochs, restoring optimal model weights) were applied during training to prevent overfitting and oscillations.

Model training was conducted in TensorFlow/Keras using the Adam optimizer with an initial learning rate of 0.001. Images were preprocessed by converting them to single-channel grayscale, resizing them to 64 × 64 pixels using area interpolation, and normalizing pixel values to [0, 1]; no online spatial or photometric data augmentations were applied. The dataset was split into 85% for training and 15% for validation. Models were trained with a batch size of 64 for up to 25 epochs. We implemented a learning rate reduction policy (ReduceLROnPlateau) that halved the learning rate (down to a minimum of 10^−5^) if validation loss failed to decrease for three consecutive epochs. Training was controlled by early stopping, which terminated optimization and restored the best model weights if validation loss did not improve over six consecutive epochs. The network was optimized using a multi-task loss objective defined as ℒ_total_ = 1.0 ⋅ ℒ_pupil_ + 0.5 ⋅ ℒ_presence_, where ℒ_pupil_ evaluates spatial coordinate regression and ℒ_presence_ measures binary cross-entropy (BCE) for eye presence. To prevent closed-eye frames from corrupting coordinate optimization, L_pupil_ is sample-weighted by a binary visibility mask m_i_ in {0, 1} (m_i_ = 1 for open eyes; m_i_ = 0 for closed eyes): $L_{\mathrm{pupil}}=\frac{1}{\sum m_{i}}\sum m_{i}\left({\left(x_{i}-\;\hat{{\left(x\right)}_{i}}\right)}^{2}+{\left(y_{i}-\hat{{\left(y\right)}_{i}}\right)}^{2}\right)$, $L_{\mathrm{presence}}=-\frac{1}{N}\sum \left(y_{\mathrm{eye},i}\log\left({\hat{y}}_{\mathrm{eye},i}\right)+\left(1-y_{\mathrm{eye},i}\right)\log\left(1-\;{\hat{y}}_{\mathrm{eye},i}\right)\right)$, where *N* is the mini-batch size, (x_i_, y_i_) and *y_eye_*_,1_ are ground-truth targets, and $\left(\hat{x_{i}}\;,\;{\hat{y}}_{i}\right)$ and ${\hat{y}}_{i}$ are model predictions. This presence-gated masking restricts coordinate loss updates strictly to open-eye frames.

### 3.4. Quantization and Deployment

After training, the model weights and activations are represented as 32-bit floating-point values. Since the ESP32-S3 lacks dedicated floating-point acceleration, INT8 quantization is applied to convert the model to 8-bit integer arithmetic. This conversion maps a continuous range of floating-point values onto the 256 discrete values representable by an 8-bit integer, significantly reducing memory usage and computational requirements. Following training, the model is exported as a TensorFlow Lite (tflite) file and subsequently converted into a C array using *xxd -i* for direct compilation into the ESP32-S3 firmware. The resulting quantized model occupies approximately 167 kB of program memory, which comfortably fits within the device’s available flash storage. In addition, a 48 kB tensor arena is allocated in external PSRAM, preserving the limited internal DRAM for the operating system, Bluetooth Low Energy (BLE) stack, image buffers, and other runtime resources required for real-time operation.

## 4. IOS Application

**Figure 7:**
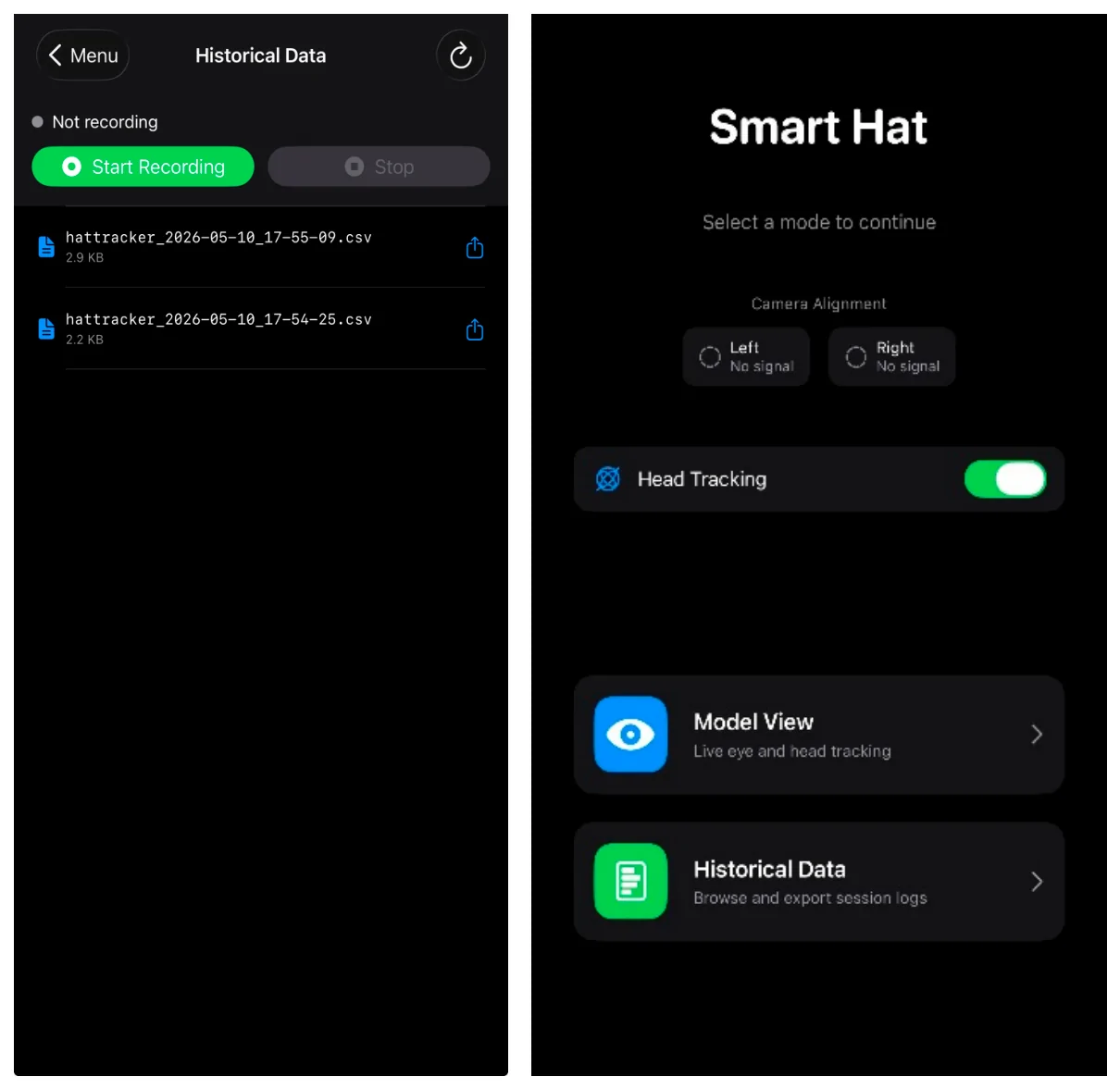
Mobile application interface for the wearable eye-tracking system. (**Left**) Main application dashboard showing camera alignment status, IMU-based head-tracking control, and access to real-time avatar visualization (Model View) and recorded session management (Historical Data). (**Right**) Historical Data interface used for session logging, storage, and export of recorded gaze-tracking data for offline analysis.

The mobile application serves as the user interface and visualization endpoint for the wearable eye-tracking microsystem. The application maintains simultaneous BLE connections to both ESP32-S3 sensing nodes and receives synchronized 26-byte data packets containing pupil coordinates, eye state information, and IMU-derived head pose measurements. Incoming packets are parsed and converted into a unified gaze stream that is used for real-time visualization and data logging. The software architecture consists of three primary layers: a BLE communication layer, a visualization layer, and a data management layer. The communication layer manages dual BLE connections and packet acquisition from the left- and right-eye sensing nodes. The visualization layer interfaces with a Unity-based rendering engine that drives the eye and neck bones of a humanoid avatar, enabling real-time replication of the user’s gaze direction and head orientation. This representation allows eye movement behavior to be monitored without storing high-bandwidth video streams, substantially reducing storage requirements while preserving the essential gaze information. A SwiftUI-based control interface provides access to both real-time monitoring and historical playback modes. Additional controls allow users to enable or disable IMU-based head tracking and monitor camera alignment status during device placement. The alignment indicators provide immediate feedback regarding eye visibility within each camera’s field of view, assisting with system calibration prior to data collection. The mobile application serves as the primary user interface for the wearable eye-tracking system and provides both real-time visualization and data management capabilities. As shown in Figure 7, the application maintains simultaneous BLE connections with the left and right sensing nodes, allowing gaze coordinates, eye state information, and head pose measurements to be received and processed in real time. The main dashboard provides camera alignment feedback and user control over IMU-based head tracking, facilitating rapid system setup prior to data collection. The application also supports a Historical Data mode, in which incoming BLE packets are recorded and stored as CSV files for later retrieval. These recordings can be replayed offline to reconstruct gaze behavior and avatar motion without requiring continuous video storage, significantly reducing memory requirements while preserving the essential eye-tracking information. Historical sessions may additionally be exported for further analysis using external tools.

## 5. Experimental Results and Model Performance

**Figure 8:**
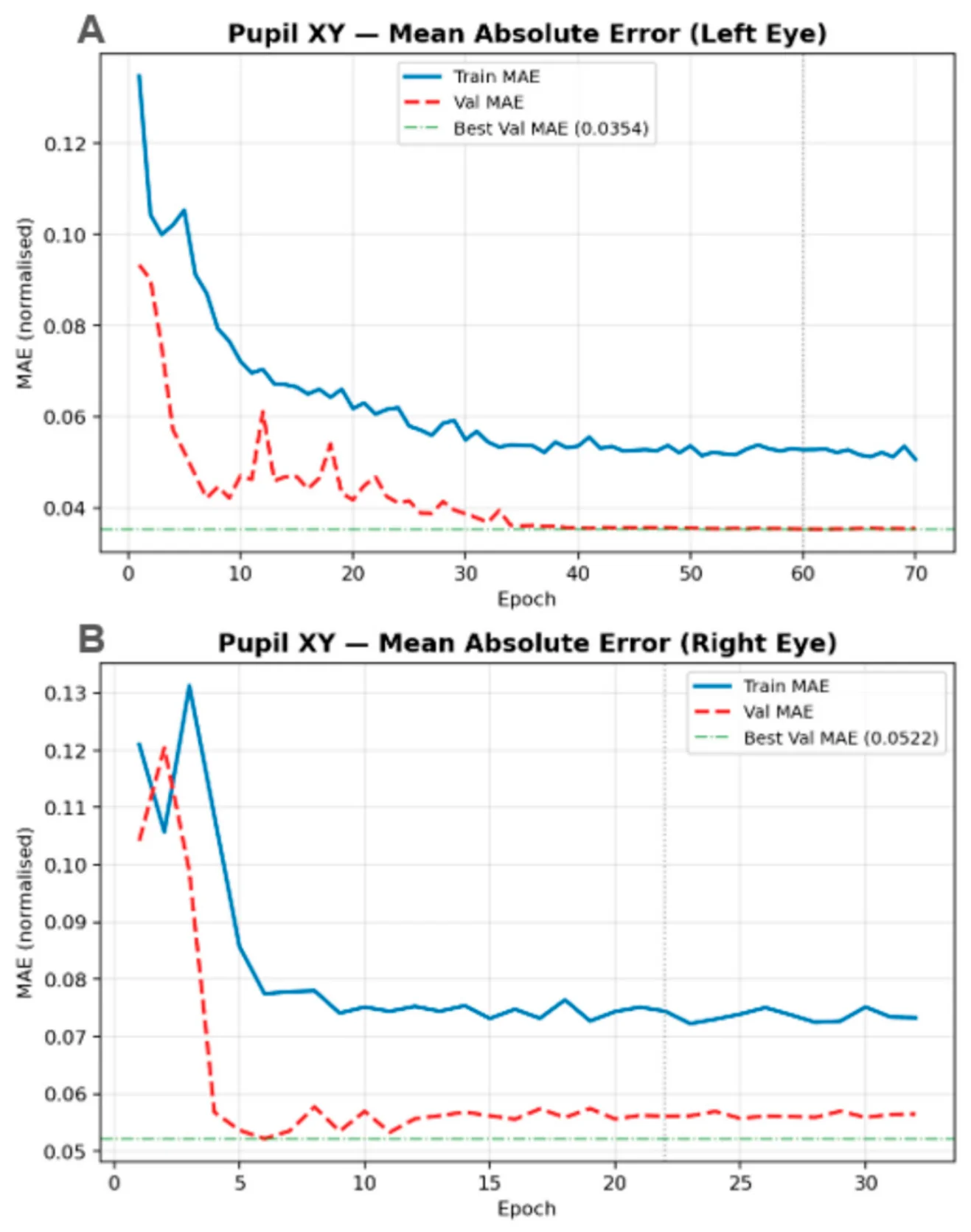
Mean Absolute Error (MAE) during training and validation of the embedded gaze estimation models: (**A**) left-eye model and (**B**) right-eye model. Both networks demonstrate convergence, with decreasing validation error across training epochs.

**Figure 9:**
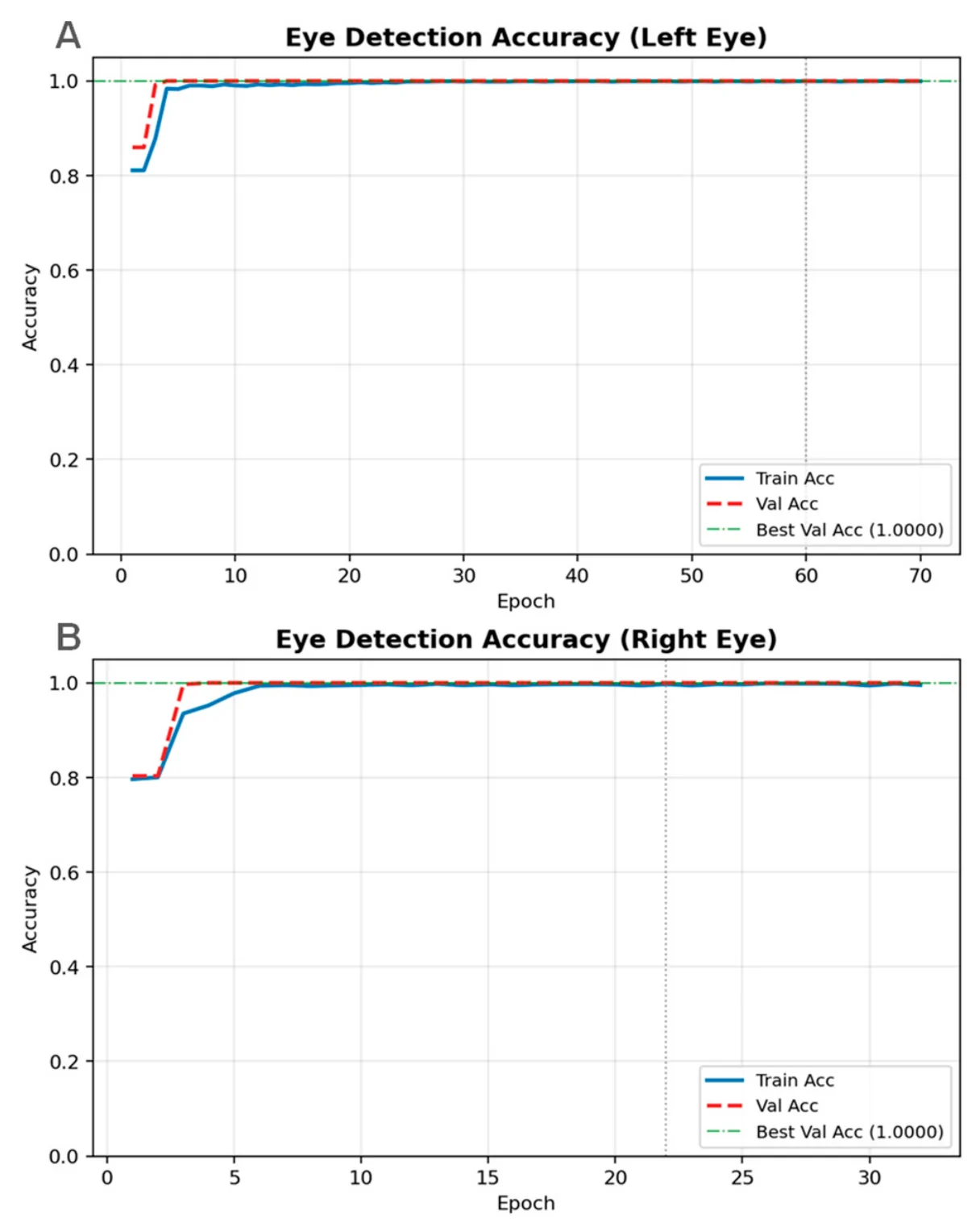
Eye presence classification accuracy during training and validation for the embedded models: (**A**) left-eye model and (**B**) right-eye model. Both networks rapidly converge to 100% validation accuracy and maintain stable performance throughout training.

**Figure 10:**
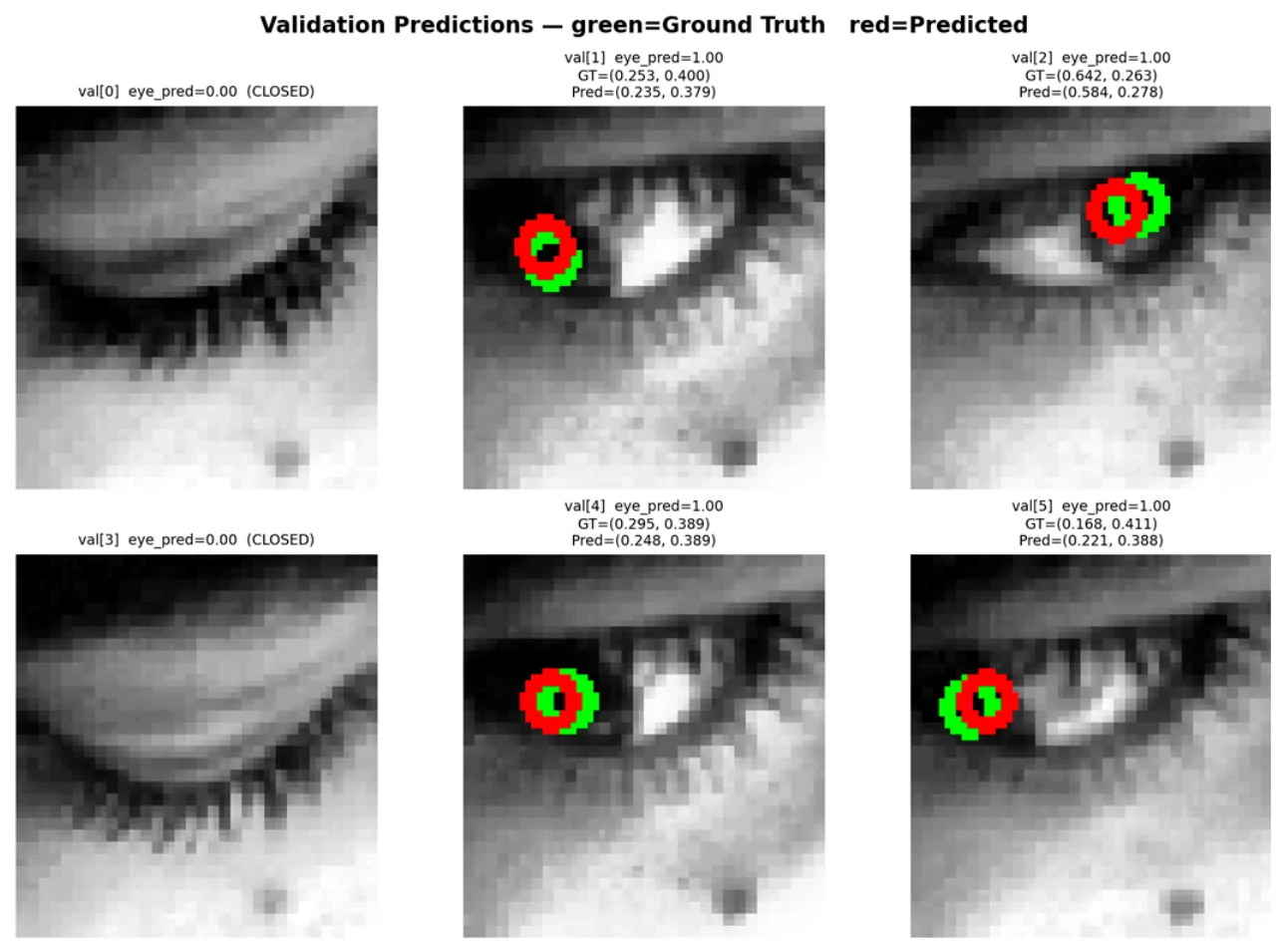
Representative validation results for the right-eye model. Predicted pupil locations (red circles) are overlaid on the ground-truth annotations (green circles).

**Figure 11:**
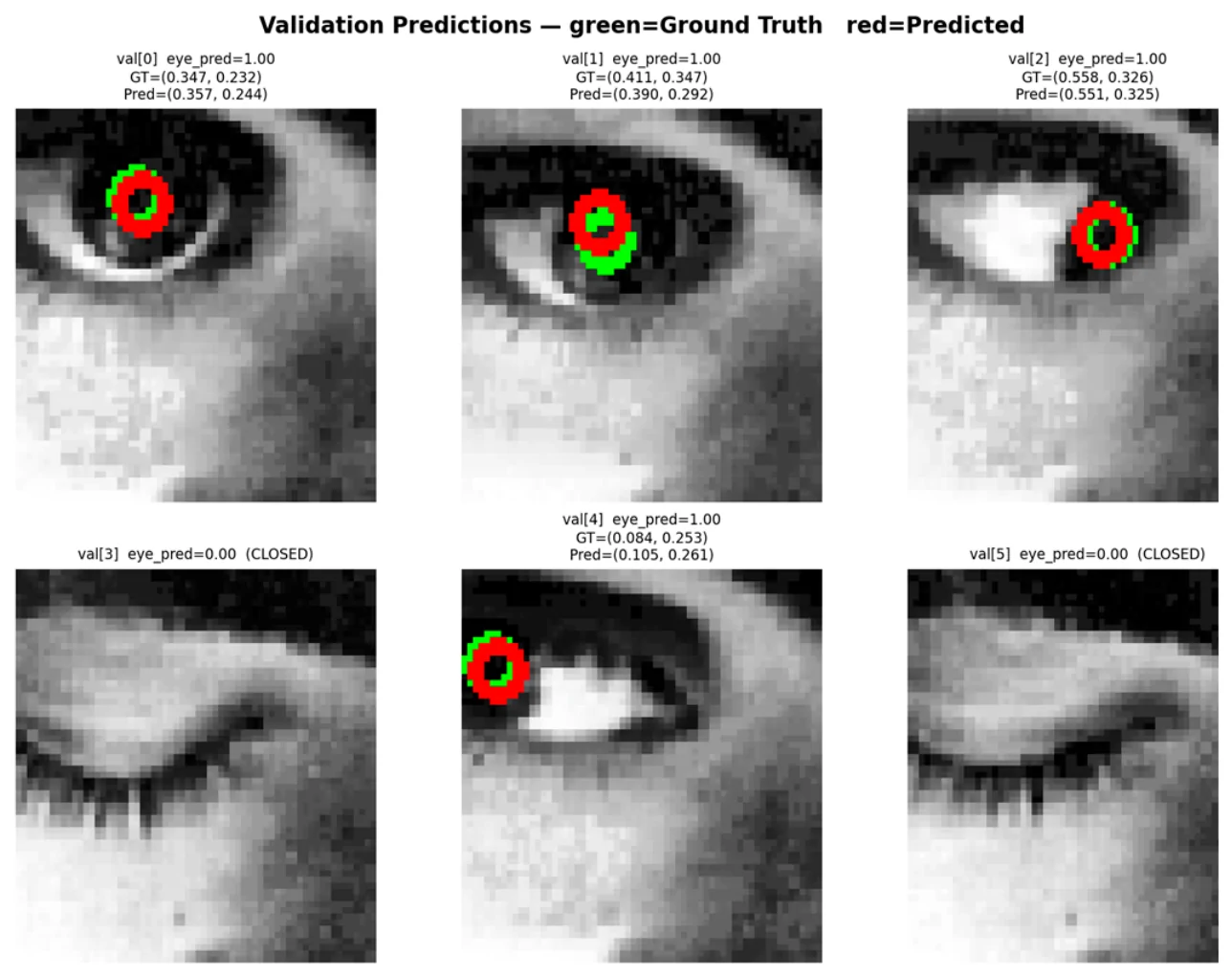
Validation predictions for the left-eye model. Predicted pupil centers (red circles) are overlaid on manually labeled ground-truth coordinates (green circles).

Both the left- and right-eye models achieved strong performance for embedded gaze estimation and eye state detection. The lowest validation MAE obtained for pupil coordinate regression was 0.0354 for the left-eye model and 0.0522 for the right-eye model, while eye presence classification reached 100% validation accuracy for both networks. As shown in Figure 8 and Figure 9, the training and validation metrics exhibit stable convergence with no evidence of significant overfitting. Although the right-eye model converged to a slightly higher regression error than the left-eye model, both models achieved sufficiently low normalized errors for reliable pupil localization. The difference is attributed to natural asymmetries in eye appearance, camera placement, eyelash occlusion, and variations in the collected training data rather than limitations of the network architecture. Figure 10 and Figure 11 present representative validation samples from the left- and right-eye models. Across a diverse range of gaze directions, including upward, downward, and lateral eye movements, the predicted pupil locations closely align with the manually annotated ground-truth coordinates. The examples demonstrate robust localization performance even when the pupil approaches the edge of the visible eye region, where perspective distortion and partial occlusion are most pronounced. Additionally, samples containing closed-eye conditions show correct suppression of pupil predictions and accurate eye state classification. These results indicate that the proposed multi-task architecture is capable of simultaneously performing pupil localization and eye presence detection using a lightweight model suitable for real-time deployment on the ESP32-S3 platform.

In initial exploratory testing using random frame-level partitioning, eye closure validation accuracy reached 100% due to contiguous frame overlap between training and validation splits. Under the strict participant/session-grouped split protocol, the model achieved an eye state classification accuracy of 95.8% on the held-out test set (Precision: 0.964, Recall: 0.952, F1-Score: 0.958). A comprehensive breakdown of classification performance across training, validation, and test splits is provided in Supplementary Table S1.

### Avatar Replay Demonstration

**Figure 12:**
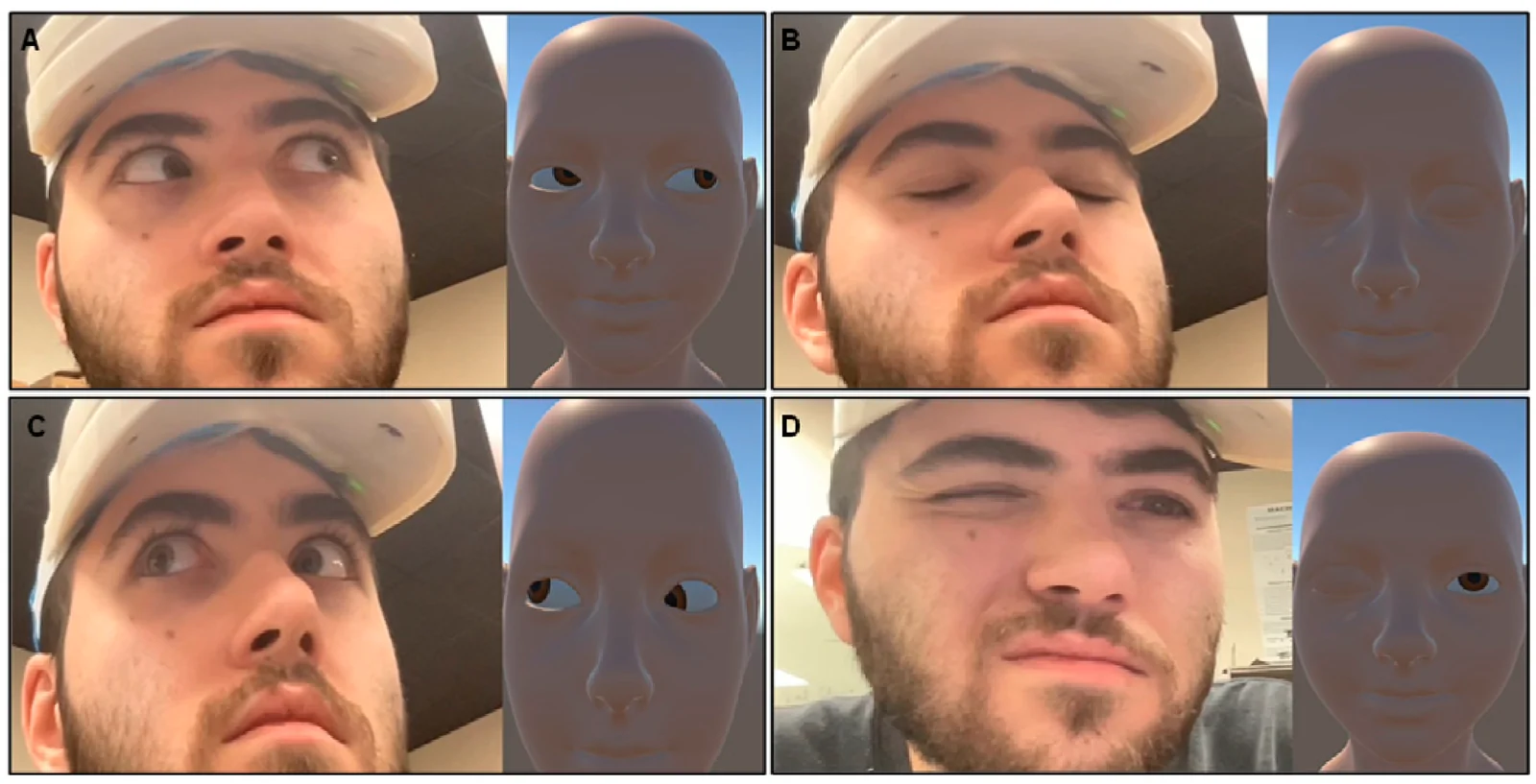
Real-time Unity-based avatar visualization driven by the wearable eye-tracking system. The avatar reproduces gaze direction and eye state information received from the embedded BLE sensing nodes. Examples include (**A**) leftward gaze, (**B**) both eyes closed, (**C**) rightward gaze, and (**D**) right-eye closure.

Figure 12 demonstrates the ability of the proposed system to reconstruct user gaze behavior in real time using BLE-transmitted pupil coordinates and eye presence predictions. The Unity-based avatar accurately reproduces both gaze direction and blink events, providing an intuitive visualization of eye activity without requiring continuous video streaming. As shown in Figure 12A,C, the avatar correctly tracks leftward and rightward gaze shifts, demonstrating the system’s ability to capture lateral eye movements. Figure 12B,D illustrate the eye presence detection capability, where the avatar accurately represents both bilateral eye closure and unilateral right-eye closure. The successful reproduction of these scenarios confirms that the embedded CNN models can simultaneously perform pupil localization and eye state classification while maintaining real-time wireless communication with the mobile application. Furthermore, the avatar-based representation provides a compact method for monitoring and replaying gaze behavior, significantly reducing storage requirements compared to video-based recording approaches.

## 6. Conclusions

This work presents a solar-assisted wearable pupil-tracking microsystem that integrates embedded AI inference, photovoltaic energy harvesting, wireless communication, and real-time visualization within a 3D-printed TPU hat platform. By relocating sensing, processing, and power management electronics to the brim of a hat, the system enables unobtrusive 2D pupil localization, eye state detection, and head pose tracking without the constraints of conventional eyewear-based solutions. The combination of INT8-quantized edge inference, solar-assisted power management, and lightweight avatar-based visualization enables long-duration operation while minimizing storage and communication requirements. While calibrated point-of-gaze estimation in physical space remains a subject for future extensions, the proposed platform provides a solid foundation for field-deployable eye motion and head-tracking applications in behavioral research, human–computer interaction, and healthcare monitoring. The timing characteristics of the pipeline were benchmarked across 1000 consecutive frames on the ESP32-S3 microcontroller and iOS application. On-device INT8 CNN inference requires 14.6 ms per frame, while preprocessing and temporal filtering take 3.2 ms and 0.8 ms, respectively. Including camera acquisition (40.0 ms), BLE transmission (18.4 ms), and mobile rendering (4.2 ms), the system achieves a total end-to-end latency of 81.2 ms and a continuous sampling rate of 22.5 FPS. A complete stage-by-stage latency breakdown is provided in Supplementary Table S2.

## Data Availability

The original contributions presented in this study are included in the article/Supplementary Materials. Further inquiries can be directed to the corresponding author.

## Supplementary Materials

The following supporting information can be downloaded at: https://www.mdpi.com/article/10.3390/mi17101183/s1, Figure S1: Physical integration of the proposed self-powered eye-tracking microsystem within the 3D-printed TPU hat; Figure S2: Flexible solar panel and its dimension; Table S1: Eye-State Classification Performance Across Dataset Splits; Table S2: System latency; Table S3: Active Hardware Power Consumption Budget; Table S4: Quantitative comparison of your proposed Solar-Assisted Hat System against standard headwear.
