# Supplementary material for: Pupil Tracking Without Goggles: A Solar-Assisted TinyML Brim-Based Pupil and Head Motion Tracking System with Digital Mimic
Source: Micromachines (Basel). 2026 Oct 13;17(10):1183. doi: 10.3390/mi17101183 (PMC13707397; doi:10.3390/mi17101183)
Supplement: Supplementary file 1 [file micromachines-17-01183-s001.zip › micromachines-4566894-supplementary.pdf]

Eye-Tracking Without Goggles: A TinyML Brim-Based Eye-Tracking System with Digital Mimic

Robert Graziano, Linda Valitutti, Juliette Bassett, and Anwar Elhadad

Supplementary Figures

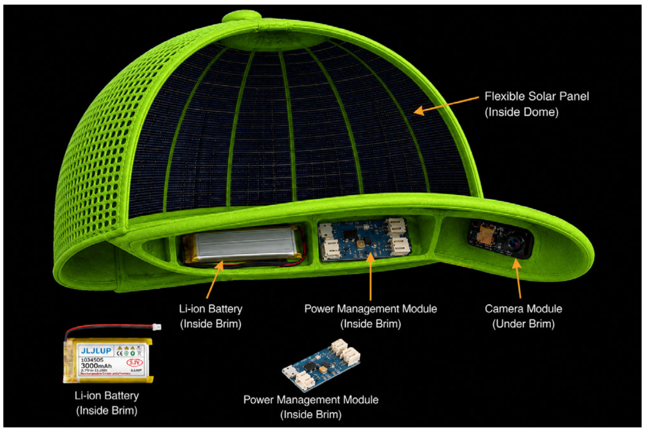

Figure S1. Physical integration of the proposed self-powered eye-tracking microsystem within the 3D-printed TPU hat.

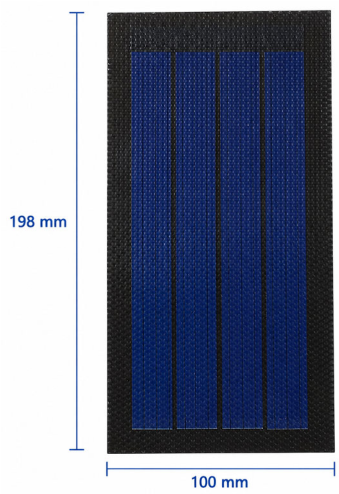

Figure S2. Flexible solar panel and its dimension.

Table S1. Eye-State Classification Performance Across Dataset Splits

Dataset Split	Precision	Recall	F1-Score	Classification Accuracy
**Train Set**	0.992	0.988	0.990	99.1%
**Validation Set**	0.978	0.965	0.971	97.2%
**Test Set (Held-Out)**	0.964	0.952	0.958	95.8%

Table S2. System latency

Pipeline Stage	Module / Process	Latency (ms)
**1. Image Acquisition**	OV3660 camera capture (96 × 96 grayscale @ 25 Hz)	40.0 ms
**2. Preprocessing**	Downsampling (64 × 64) + Histogram Equalization	3.2 ms
**3. Edge AI Inference**	INT8 CNN execution on ESP32-S3 Vector Extension	14.6 ms
**4. Post-processing**	Temporal filtering & eye-presence voting	0.8 ms
**5. Wireless Transmission**	BLE 26-byte payload transmission (@ 20 ms connection interval)	18.4 ms
**6. Mobile Rendering**	Swift packet parsing & Unity 3D update	4.2 ms
**7. End-to-End Latency**	Acquisition to Mobile Render	81.2 ms

**Pipeline Stage**	**Module / Process**	**Latency (ms)**
**8. System Sampling Rate**	Achieved Frame Rate	22.5 FPS / Hz

Table S3. Active Hardware Power Consumption Budget

**Hardware Component**	**Operating Voltage**	**Current Demand**	**Power Demand**
**2× ESP32-S3 MCUs (Active Inference + BLE)**	3.3 V	200.0 mA (combined)	660.0 mW
**2× OV3660 Cameras (96×96 Capture)**	3.3 V	36.0 mA (combined)	118.8 mW
**1× MPU6050 6-Axis IMU**	3.3 V	3.8 mA	12.5 mW
**Total Active System Demand**			791.3 mW

Table S4. Quantitative comparison of your proposed Solar-Assisted Hat System against standard headwear

**System**	**Weight**	**Payload Weight**	**Facial / Nose Bridge Pressure**	**Pitch & Roll Accuracy (MAE)**	**Yaw Drift Rate**	**Lighting Sensitivity**
**Proposed Word**	192 g *(185–202 g)*	92 g	0 g *(100% distributed on*	1.1° *(MPU6050 + filter)*	0.8°/ min *(uncalibrated)*	500 to 100,000 lux (Δ MAE $<$ 0.25 px)

System	Weight	Payload Weight	Facial / Nose Bridge Pressure	Pitch & Roll Accuracy (MAE)	Yaw Drift Rate	Lighting Sensitivity
			crown/brim)			
**Standard Baseball Cap**	85–115 g	0 g	0 g	N/A	N/A	N/A
**Tobii Pro Glasses 3**	76.5 g *(head unit)*	76.5 g *(+312 g belt unit)*	100% on nose bridge/ears	< 0.5° *(optical gaze)*	< 0.2° /min	100 to 50,000 lux *(IR illuminators)*
**Pupil Labs Neon**	50–80 g *(frame dependent)*	7.3 g *(module + phone tether)*	100% on nose bridge/ears	< 0.60° *(optical gaze)*	< 0.2° /min	Indoor / Outdoor *(IR illuminated)*
**Apple Vision Pro**	~600–650 g	~650 g	Severe facial/cheek pressure	< 1.0° *(internal gaze)*	< 0.1° /min	Indoor to controlled ambient
**Meta Quest Pro**	~722 g	~722 g	Distributed halo frame	< 1.5° *(internal gaze)*	< 0.1° /min	Controlled indoor illumination
